# *Prunella vulgaris* seed oil alleviates cancer related fatigue through hypothalamic inflammation and CRH regulation

**DOI:** 10.1038/s41538-025-00541-5

**Published:** 2025-09-24

**Authors:** Changyu Wu, Qing Hu, Mingzhi Han, Yuhan Peng, Chengyu Zhang, Zhangjie Wu, Ruiyi Liu, Shan Xing, Ying Yin, Mária A. Deli, Hailou Zhang, Gang Chen

**Affiliations:** 1 Interdisciplinary Institute for Personalized Medicine in Brain Disorders, & Guangdong-Hong Kong-Macao joint Laboratory of Traditional Chinese Medicine on Brain-Peripheral Homeostasis and comprehensive Health, Jinan University, Guangzhou, PR China; 2 Zhuhai Institute of Jinan University, Zhuhai, PR China; 3 Department of Food Science and Engineering, Jinan University, Guangzhou, Guangdong PR China; 4 Danzhai County People’s Hospital, Danzhai, Guizhou PR China; 5 Institute of Biophysics, HUN-REN Biological Research Centre, Szeged, Hungary

**Keywords:** Diseases of the nervous system, Neuroimmunology, Cancer

## Abstract

Cancer-related fatigue (CRF) is a common symptom in cancer patients, affecting their quality of life. *Prunella vulgaris* and its seed oil (PVSO) are edible-medicinal foods with bioactive compounds that may alleviate CRF. The present study aimed to test the effects of PVSO on CRF and underlying mechanisms. We found PVSO improved fatigue-like behaviors in both LPS-induced and tumor-bearing models. In the breast cancer tumor-bearing animals, PVSO reduced tumor growth, and suppressed serum and hypothalamic inflammatory cytokines (*TNF-α, IL-1β*, and *IL-6*) and NF-κB signaling. Furthermore, the hypothalamic corticotropin-releasing hormone (CRH) expression and serum CRH/cortisol levels dampened in tumor-bearing animals were normalized by PVSO. Our results suggest that PVSO exhibits potent anti-CRF effects likely via attenuation of cancer-induced hypothalamic inflammation and subsequent normalization of the dysregulated HPA axis. PVSO may be developed as a functional food for breast cancer-related fatigue.

## Introduction

Breast cancer is a significant worldwide public health issue, with a global occurrence rate of 24.2% among female cancer patients^1^. It is the most common type of cancer in terms of both new cases and deaths. Thanks to the comprehensive research and the development of innovative strategies for prevention and treatment, the death rate has significantly decreased^2^. Moreover, with appropriate medical intervention, the long-term survival rate of patients can reach 90%^3^. Despite these improved survival rates, empirical evidence indicates that cancer patients experience a range of mental health problems, such as fatigue, depression, anxiety^4^, and insomnia^5^. Cancer-related fatigue (CRF), a symptom of cancer cachexia, significantly affects the quality of life of patients and is particularly severe in female patients. This fatigue is more persistent and severe than general fatigue and may directly affect the outcome of treatment. CRF is not only about the patient’s physical health, but also affects the patient’s psychological status and quality of life.

Fatigue, classified as a condition of suboptimal health, typically presents as both physiological and psychological exhaustion^6^. The current treatment methods for CRF include aerobic exercise^7^, psychosocial intervention^8^, psychosomatic intervention and drug intervention^9^, as well as appropriate supplementation of dietary nutrients^10^. There are certain toxic side effects with drug therapy, including possible post-treatment exacerbation of CRF, tumor growth, and increased cardiovascular risk^11,12^. Non-pharmacologic interventions generally confer low risk, but has only moderate efficacy. Currently, safe and effective treatment of CRF is still a challenge. There is a lack of clarity regarding the physiologic mechanisms of CRF^1,13^. Increasing number of studies have revealed biological mechanisms such as inflammation, hypothalamic-pituitary-adrenal (HPA) dysfunction and skeletal muscle depletion^14^. Inflammation has a crucial role in the development and progression of CRF^15^. It is known that during the course of cancer and certain cancer treatments, immune cells and cancer cells themselves produce pro-inflammatory substances that induce CNS inflammation and activate the HPA axis, contributing to CRF^16,17^. However, how tumor-induced immune disorders lead to HPA axis dysregulation underlying CRF remains unclear.

*Prunella vulgaris* (PV), a medicinal and food plant, is commonly used to intervene headaches, vertigo, goiter and breast enlargement^18,19^. The main components of PV include terpenoids, flavonoids, phenolic acids, sterols, polysaccharides and volatile oils^20,21^, and possesses a diverse range of pharmacological properties, including anti-inflammatory, antiviral, and antineoplastic effects^22,23^. Recently, there has been a growing interest in the effects of PV extract and its active components on inhibitory effects on tumor growth. Several studies have showed that PV and its natural active ingredients slow the growth of tumor cells through multi-pathway targeting, and at the same time have little cytotoxicity to normal cells^24–26^. *Prunella vulgaris* seed oil (PVSO) can be used as a novel resource for medicinal food. PVSO extracted by supercritical CO_2_, contains a variety of active ingredient substances shared with PV, and are rich in unsaturated fatty acids and phenolics, which make it potent for mitigation of CRF.

Here we aimed to test the role of PVSO in alleviation of CRF and the underlying mechanism, focusing on the specific role of the neuroinflammation in the hypothalamus in HPA dysregulation. We demonstrated that PVSO alleviated fatigue-like behavior in two fatigue models, and its improvement of breast-cancer related fatigue may be mediated by attenuating the hypothalamic inflammation induced by infiltration of tumor, and subsequent restoring corticotropin-releasing hormone (CRH) and cortisol levels. These findings may provide a scientific basis for the application of PVSO as a functional food ingredient in alleviating breast cancer-related fatigue.

## Results

### Dose-effect relationship of PVSO on relief of lipopolysaccharide (LPS)-induced fatigue

**Fig. 1:**
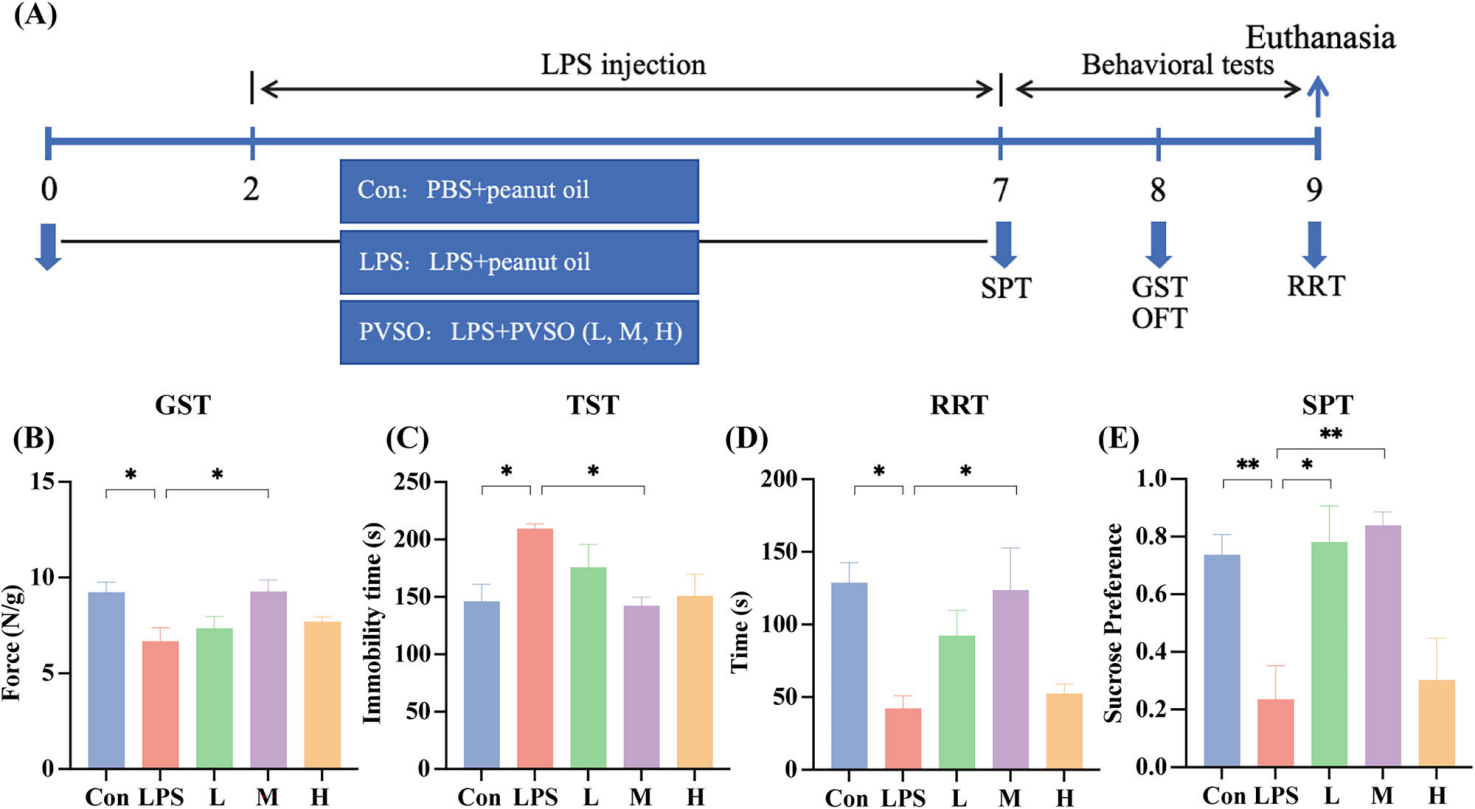
Animal experiment and behavioral tests in the LPS-injection model. **A** Experimental animal procedures: from day 0, mice were given three doses of *Prunella vulgaris* seed oil (PVSO) at low (L, 0.0104 g/kg), medium (M, 0.0208 g/kg) and high (H, 0.0312 g/kg) doses, from day 2 to day 7 in LPS model mice and intervention group mice were injected intraperitoneally with lipopolysaccharide (LPS) and control mice (Con) were injected with saline. Mice in the medium PVSO dose showed significant improvement in (**B**) Grip strength magnitude in the grip strength test (GST). **C** The immobility time of the tail suspension test (TST). **D** Latency time of the rod rotation test (RRT), and **E** Sucrose preference index of Sucrose preference test (SPT). Data are presented as mean ± SEM, *P < 0.05, **P < 0.01 by One-way ANOVA (n = 6 per group).

LPS can cause peripheral and central inflammation, which presumably in turn alters HPA axis function to trigger fatigue, and has been widely used as a fatigue model^27–29^. Therefore, to assess the anti-fatigue potential of PVSO and to investigate its dose-response relationship, we first performed a dose-ranging pharmacological assessment under the LPS-induced fatigue model. Behavioral results indicated that PVSO had significant anti-fatigue and antidepressant effects at a dose of 0.13 g/kg (equivalent to 0.0208 g/kg based on drug concentration) (Fig. S1A–D). Based on this result, 0.0208 g/kg was designated as the intermediate dose for subsequent experiments, and we similarly evaluated the anti-fatigue effects of low, intermediate, and high doses of PVSO under the LPS model. Mice were administrated with LPS for 5 days, and PVSO was given for 7 days, starting from two days before the LPS administration (Fig. 1A). Compared to Con group, the LPS-treated mice exhibited decreased grip strength, increased rotarod test (RRT) latency time and tail suspension test (TST) immobility time (*p* < 0.05). The PVSO medium group at a dose of 0.0208 g/kg showed significant improvement in grip strength, TST immobilization time, and RRT latency time, compared to the LPS group (*p* < 0.05). In addition, the medium dose of PVSO also exhibited an increased preference for sucrose compared to the LPS group (*p* < 0.01) (Fig. 1B–E). In contrast, PVSO at lower dose or higher dose hardly improved the above behaviors. These data indicated that PVSO at a dose of 0.0208 g/kg could alleviate LPS-induced fatigue as well as depressive-like behavior, which was used in the subsequent experiments.

During the experiment, we monitored changes in the animals’ body weight. After LPS intervention, the body weight of animals in all groups decreased, with the most severe decrease of 4.00% in the LPS group. However, no deaths occurred, and the body weights of the mice gradually recovered in the later stages of the experiment (Fig. S2A). In the open field test (OFT), all LPS-treated mice showed the tendency to reduce locomotor activity after LPS administration (Fig. S2B). However, this reduction was not statistically significant, and exploratory behavior was preserved in all groups, indicating no obvious disease-induced reduction in activity. Notably, mice treated with moderate-dose PVSO (0.0208 g/kg) spent more time in the central region than the LPS group, suggesting a potential anxiolytic-like effect (Fig. S2C). These results indicate that LPS induced a fatigue-like state without triggering classic disease-related behaviors, while PVSO, particularly at moderate doses, improved the behavioral deficits without exacerbating systemic disease.

### PVSO slowed down the cancer progression on 4T1-induced breast cancer mice

**Fig. 2:**
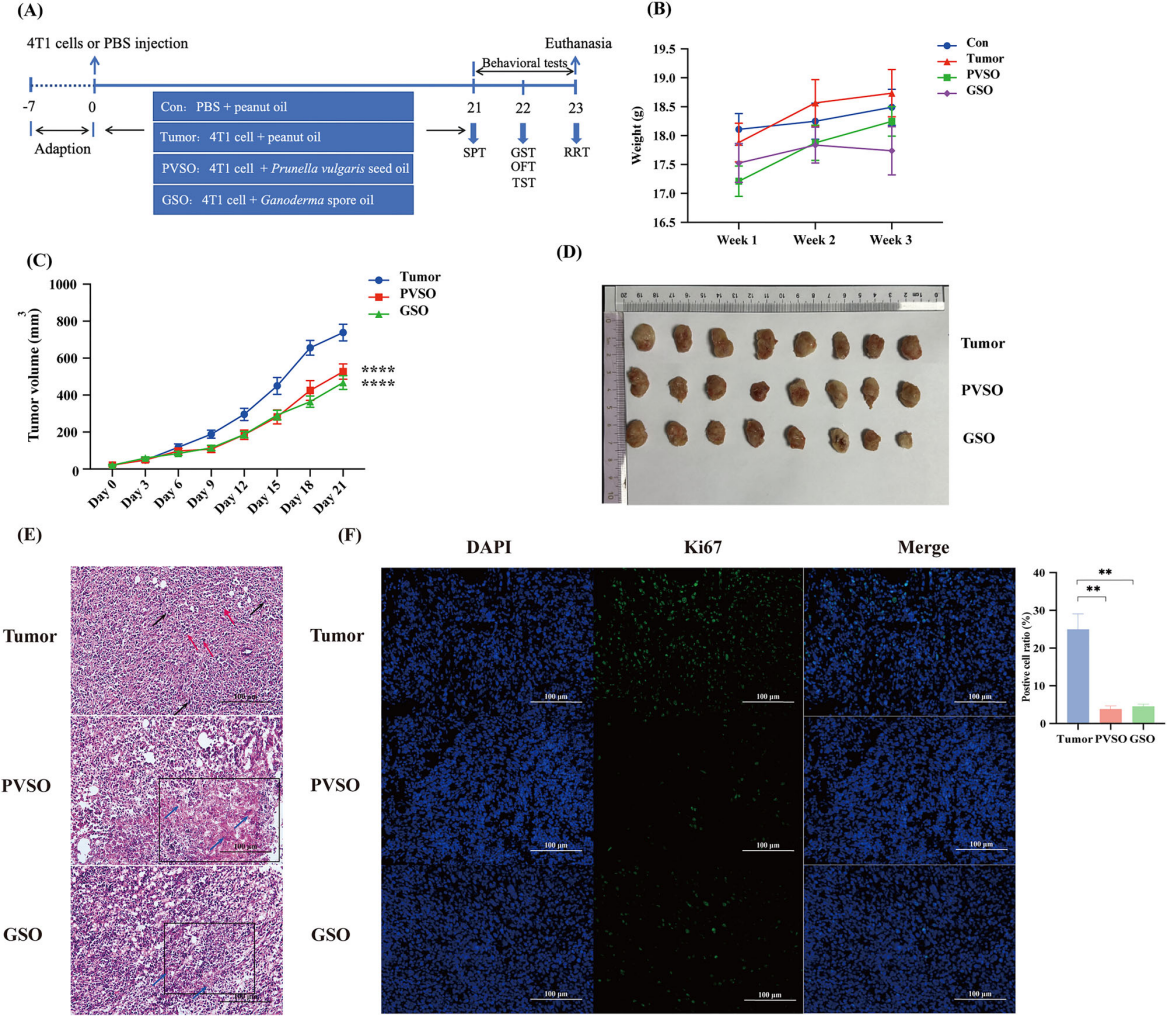
Effects of PVSO on cancer progression in mice with 4T1 breast cancer tumors. **A** Experimental animal procedures: on day 0, mice were injected subcutaneously on the right side with 1 × 10^5^ 4T1 tumor cells or PBS. From day 1 to day 21, mice were gavaged once daily with *Prunella vulgaris* seed oil (PVSO) and *Ganoderma spore* oil (GSO) or drinking water (Tumor). On day 7, mice could palpate the tumor. Mice were behaviorally tested on day 21 and then euthanized. Mice were weighed weekly and tumor volumes were measured on days 0, 3, 6, 9, 12, 15, 18 and 21. **B** Weekly body weight of mice. **C** Tumor volume changes over time. Both PVSO and GSO showed improvement. **D** Size of tumors removed after euthanasia. **E** Sections of tumor tissue stained with hematoxylin and eosin. The cytoplasm is eosinophilic (indicated by the red arrow), Large areas of necrosis were observed in the cancerous tissue of the PVSO group and GSO group (black box), with most of the necrotic cells and disintegrated stroma fused into a blurred, granular, unstructured red-stained substance (blue arrow). **F** Ki67-immunofluorescence stained-tumor tissue sections. Data are presented as mean ± SEM, Two-way ANOVA (**B**, **C**), otherwise use One-way ANOVA, **P < 0.01, ****P < 0.0001 (n = 8–10 per group).

A mouse model of breast cancer was constructed by subcutaneous inoculation of 4T1 breast cancer cells. We examined the effect of PVSO on cancer progression in mice with breast cancer, using subcutaneous inoculation of 4T1 breast cancer cells in female Balb/c mice (Fig. 2A). There were no significant differences in body weights, which included tumor’s weights, among the mice in each group over the three-week period. The GSO group, serving as a positive control group for anti-CRF, appeared not to gain body weights after the second week, whereas the Tumor and PVSO group appeared to gain some body weights from week 1 to week 3 (Fig. 2B). The tumor volume growth curves revealed that starting from the third day, distinct variations in tumor volume were observed among the groups. Specifically, the mice in the Tumor group exhibited rapid tumor growth, distinguishing them from the other two groups. Upon completion of the experiment, the tumors were removed from the mice and measured in terms of weight. The results indicated that the administration of PVSO and GSO led to a reduction in tumor weight as compared to the Tumor group (*p* < 0.01). There was no discernible difference in tumor weight between the PVSO and GSO groups (Fig. 2C, D).

Under light microscopy, the pathological examination of the Tumor group revealed that the cancerous tissue was arranged in nests, with cancer cells appearing round in shape. There was significant cellular atypia, with eosinophilic cytoplasm (indicated by the red arrow), diverse nuclear morphology, and numerous mitotic figures (indicated by the black arrow). Compared with the Tumor group, the PVSO and GSO groups exhibited extensive necrotic areas (black box) within the cancerous tissue, with most necrotic cells and disintegrated stroma fusing into a blurred, granular, unstructured red-stained substance (blue arrow) (Fig. 2E). Furthermore, the necrotic cells in these groups were predominantly heavily pigmented with solid nuclei. The Tumor group of mice exhibited a substantial quantity of Ki67-immunopositive cells, while the PVSO and GSO groups displayed a noteworthy decrease in Ki67-positive cells inside the tumor tissues. When comparing the Tumor group to both the PVSO and GSO groups, it was found that both PVSO and GSO groups had a significant reduction in the Ki67 positive rate (Fig. 2F).

### PVSO improved fatigue behavior in tumor-bearing mice

**Fig. 3:**
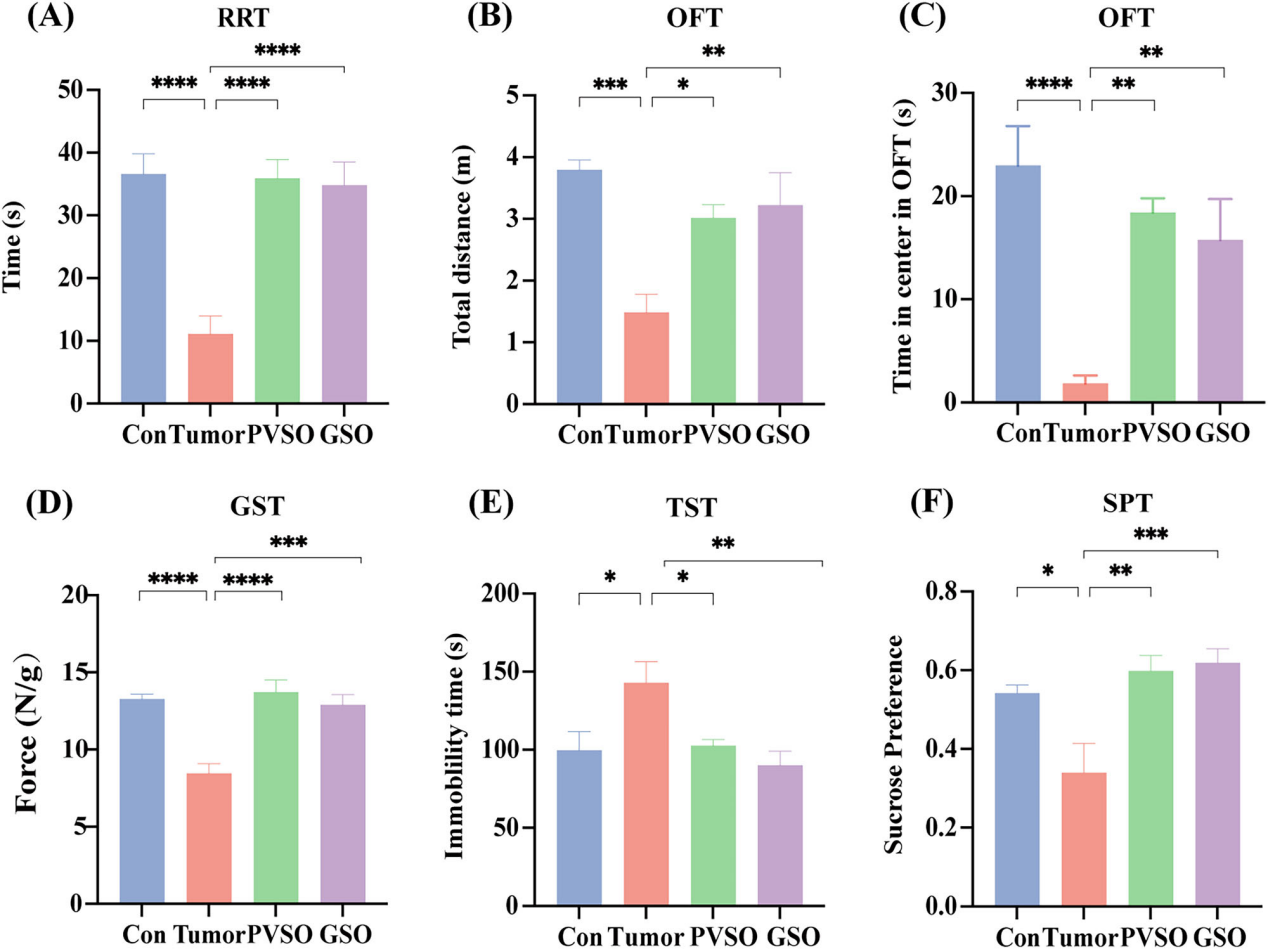
Effects of PVSO on fatigue and depression-like behavior in 4T1 tumor-bearing mice. **A** Latency time of rotating rod test (RRT) after 21 days of PVSO, GSO or control intervention. **B** Total distance travelled and **C** Time spent in the center of open field test. **D** Grip strength magnitude in the grip strength test (GST). **E** Immobility time of tail suspension test (TST). **F** Sucrose preference index of Sucrose preference test (SPT). Data are presented as mean ± SEM, *P < 0.05, **P < 0.01, ***p < 0.001 by One-way ANOVA (n = 8–10 per group).

Following 21-days treatment on the tumor-bearing mice, mice were subjected to behavioral tests. In comparison to the Tumor group, mice within the PVSO and GSO groups manifested a statistically significant elevation in latency during the RRT (Fig. 3A) (*p* < 0.0001), along with a notable increase in grip strength for both groups (Fig. 3D). In the OFT test, there was a significant augmentation in the time spent at the center and the traversed distance in the PVSO and GSO groups (Fig. 3B, C). In the TST test, the PVSO group had a significant reduction in immobilization time compared to the Tumor group (Fig. 3E). Additionally, mice in the PVSO group demonstrated an increased preference for sucrose (Fig. 3F). In sum, the intervention of PVSO and GSO improved fatigue-like behaviors, and showed antidepressant effects.

### Breast tumor selectively upregulated inflammation levels in the hypothalamus, ameliorated by PVSO

**Fig. 4:**
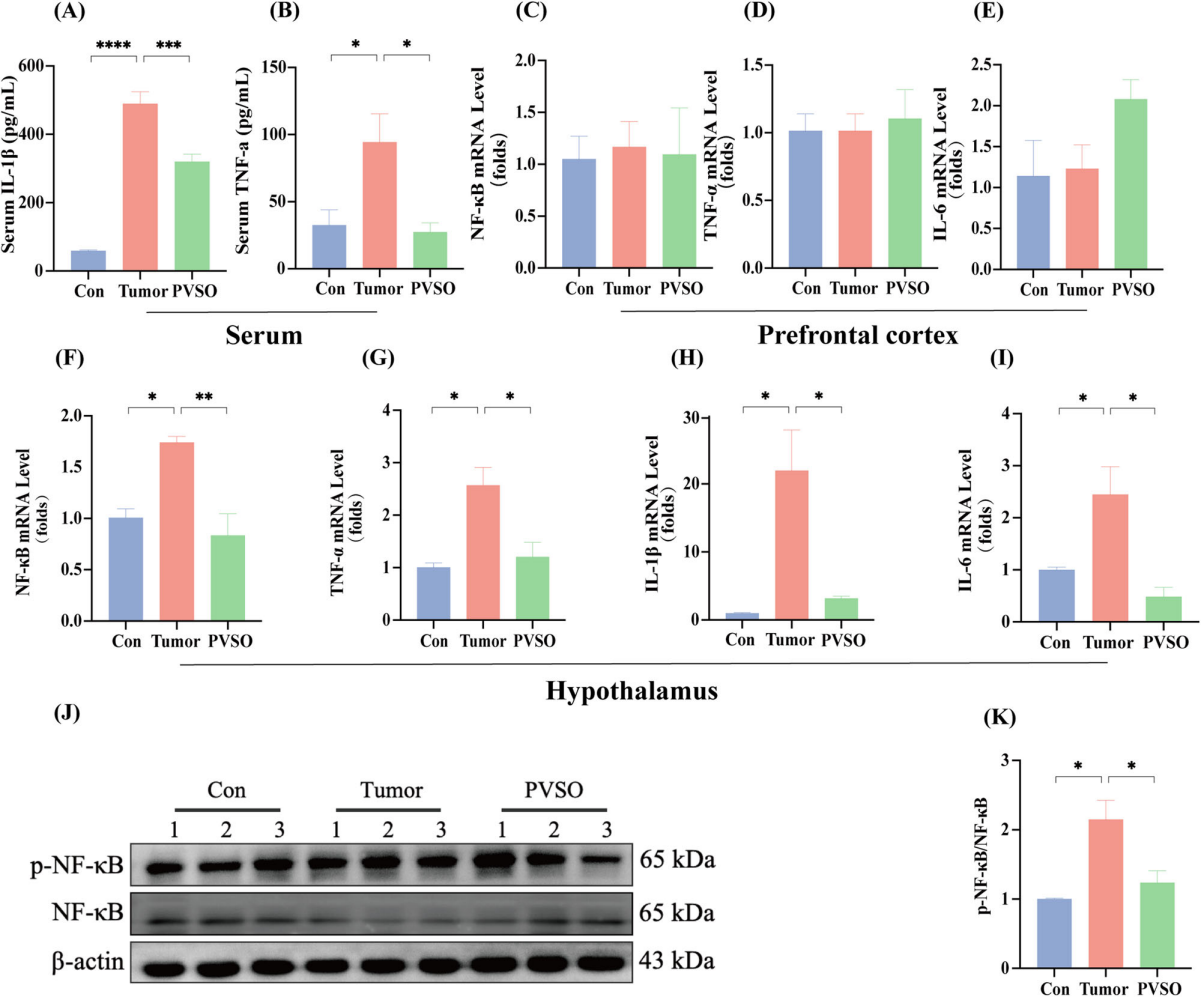
Effects of inflammation by PVSO in 4T1 tumor-bearing mice. **A**, **B** Serum TNF-a and IL-1β levels (n = 4). **C**–**E** Inflammatory factor expression levels in the prefrontal cortex (n = 4). **F**–**I** Inflammatory factor expression levels in the hypothalamus. **J**–**K** Representative western blots of NF-κB (n = 3). Data are presented as mean ± SEM, *P < 0.05, **P < 0.01, ***p < 0.001 by One-way ANOVA (n = 3–4 per group).

To test if the breast tumor induces inflammation in the brain in a region-specific manner, we compared the inflammation in two limbic regions implicated in fatigue, the hypothalamus and the prefrontal cortex (PFC). In serum, the levels of TNF-α and IL-1β were notably higher in the Tumor group in comparison to the Con group, and there was a significant downregulation in the PVSO group (Fig. 4A, B). In the PFC, the gene expressions of *TNF-α*, *IL-6*, *IL-1β* and *NF-κB* signaling did not display significant changes (Fig. 4C–E). In contrast, in the hypothalamus, the expressions of *TNF-α*, *IL-6*, *IL-1β* and *NF-κB* were elevated in the Tumor group of mice (Fig. 4F–I), which were all reversed by the treatment of PVSO group (*p* < 0.05). Consistently, the protein expression of total NF-κB and phosphorylated NF-κB was significantly upregulated by tumor, which was suppressed after administration of PVSO (Fig. 4J–K) (*p* < 0.05).

### Effects of PVSO on the expression of CRH and associated HPA signaling in the hypothalamus of 4T1 tumor-bearing mice

**Fig. 5:**
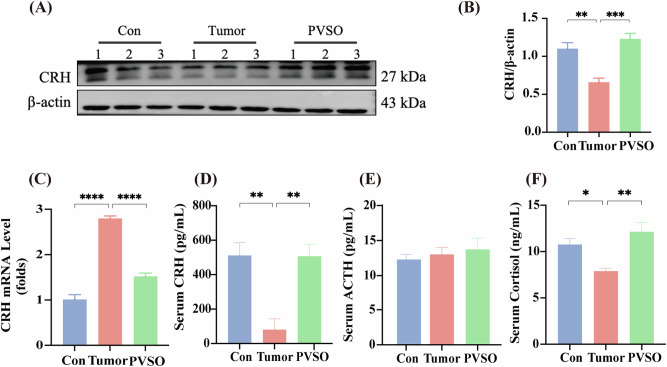
Effects of PVSO on HPA axis in 4T1 tumor mice. **A**, **B** Representative western blots of CRH. **C** Expression levels of *CRH* in the hypothalamus. **D** Serum CRH levels. **E** Serum cortisol levels. **F** Serum adrenocorticotropic Hormone (ACTH) levels. Data are presented as mean ± SEM, *P < 0.05, **P < 0.01, ***p < 0.001, ****p < 0.0001 by One-way ANOVA (n = 3–4 per group).

We further investigated CRH expression in the hypothalamus and HPA axis following the tumor and drug treatment (Fig. 5A, B). The peptide expression of CRH in the hypothalamus in the Tumor group was markedly decreased, compared to Con group. In contrast to the Tumor group, the expression of CRH in the PVSO group was upregulated (*p* < 0.05). However, *CRH* gene expression in the hypothalamus of the tumor group was significantly increased, while PVSO significantly reduced *CRH* expression levels (*p* < 0.0001) (Fig. 5C). Concurrently, Serum levels of CRH, adrenocorticotropic hormone (ACTH), and cortisol were also measured. Mice in the Tumor group presented lower concentrations of CRH and cortisol in serum, while PVSO intervention resulted in a significant elevation (Fig. 5D, F) (*p* < 0.01). Nevertheless, there was no difference in ACTH across the groups (Fig. 5E).

### Correlation analysis between behavioral indices, CRH and cortisol levels and inflammation

**Fig. 6:**
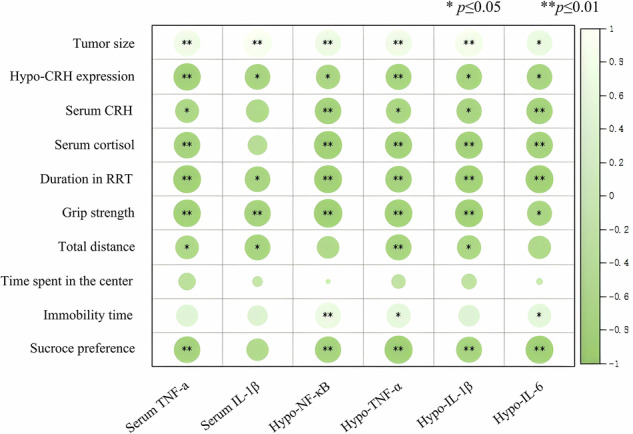
Correlation analysis between behavioral indices, CRH and cortisol levels and inflammation. Spearman’s rank correlation coefficient (r) was computed, where r ranges from −1 (perfect negative correlation) to 1 (perfect positive correlation). An absolute r value ≥ 0.5 indicates a moderate or stronger association. Statistical significance is marked by asterisks: *P < 0.05, **P < 0.01, and no asterisk indicates *P* ≥ 0.05 (The shade of the color indicates the strength of the correlation, with white representing a positive correlation and green representing a negative correlation, and the darker the color the stronger the correlation. The size of the circle indicates significance, the larger the size, the higher the significance).

Spearman correlation analysis revealed that tumor size was significantly positively correlated with both peripheral and hypothalamic inflammation. In contrast, hypothalamic CRH expression was negatively correlated with inflammation. Fatigue-related behavioral phenotypes, including grip strength and latency of RRT, also showed a negative correlation with inflammation (Fig. 6).

## Discussion

The objective of this study was to elucidate the anti-fatigue properties of PVSO in breast tumor and its underlying mechanisms of action. The anti-fatigue efficacy of PVSO was systematically evaluated and confirmed using both LPS-induced and breast cancer bearing models. Tumor induced peripheral inflammation and hypothalamic-specific inflammatory responses, along with a significant decrease in CRH expression and cortisol levels, indicating a dysfunction of the HPA axis. PVSO improved both tumor progression and inflammation in the peripheral and hypothalamus and restored the CRH expressions. These findings suggest PVSO exerts anti-CRF effects by normalization of hypothalamic CRH levels and hence HPA axis, likely via reducing the hypothalamic inflammatory signaling.

LPS-induced inflammation and tumor progression have both been linked to fatigue, suggesting a potential shared pathogenic mechanism underlying CRF^30,31^. Both trigger the release of pro-inflammatory cytokines (TNF-α, IL-1β and IL-6), activate microglia, increase blood-brain barrier permeability, and disrupt neurotransmitter balance (5-HT, DA, GABA), all contributing to neuroinflammation-driven fatigue^32^. In our study, mice subjected to either LPS administration or tumor cell inoculation exhibited remarked fatigue-like behaviors, as evidenced by reduced grip strength and shortened RRT duration. The use of LPS is especially useful to screen for the optimal dose range for CRF, as the latter process is much more complicated and time-consuming. In this study, PVSO showed similar anti-fatigue effects in LPS model and tumor-bearing models, supporting its therapeutic potential in targeting inflammation-driven fatigue. It is worth to note that, however, there exist some differences in peripheral and central inflammation between these two models, indicated here with the region-specific inflammation in the tumor-bearing model.

This study explores the potential of PVSO as a therapeutic intervention for CRF by targeting inflammation-driven mechanisms in the central nervous system. Tumor-bearing mice exhibited significantly elevated levels of TNF-α and IL-1β, reinforcing the role of systemic inflammation in driving fatigue-like behaviors^33,34^. Correlation analysis confirmed a strong association between inflammatory markers and fatigue severity, supporting inflammation as a central contributor to CRF. Evidences indicate that persistent peripheral inflammation can influence the central nervous system (CNS), leading to neuroinflammation and subsequent behavioral symptoms such as fatigue and depression^35,36^. In this study, we provide evidence that tumor-induced systemic inflammation activates NF-κB signaling in the hypothalamus, leading to upregulation of key pro-inflammatory genes (*IL-1β*, *TNF-α* and *IL-6*) within the CNS. Crucially, PVSO treatment significantly reduced systemic inflammation, suppressed NF-κB activation inflammatory cytokine expression in the hypothalamus.

Previous studies have primarily focused on the anti-inflammatory and anti-tumor effects of PV as a whole herb, while this study is the first, for the best of our knowledge, to show the anti-tumor effects of the seeds of PV. PV seeds retain the active components of the whole herb and can serve as a safe dietary supplement. The PVSO utilized in this study was extracted via supercritical fluid extraction, a method that preserves its bioactive integrity, including a high concentration of polyunsaturated fatty acids (PUFAs), with omega-3 unsaturated fatty acids accounting for more than half of the total mass. PUFAs are critical for various biological and metabolic processes, and plant-derived PUFAs have been reported to exhibit anti-inflammatory properties^37,38^, suppress the synthesis of pro-inflammatory cytokines in response to LPS stimulation^39,40^ and attenuate NF-κB activation^41^. Our study provides new perspectives on CRF treatment, using bioactive lipids extracted from dietary recourse.

Few previous studies have explored the regional sensitivity of neuroinflammation in CRF. Our study provides new insights by demonstrating that tumor-induced activates neuroinflammatory signaling in a brain-selective manner, particularly in the hypothalamus, while the PFC remains largely unaffected. The PFC plays a significant role in modulating behaviors related to fatigue, including cognitive and emotional processing, decision-making, and the regulation of physical activity^42^. In the context of CRF, the PFC is thought to influence how individuals perceive and cope with fatigue, potentially by integrating sensory and motivational signals^43^. Its ability to regulate effort and energy expenditure makes it a key region for managing the subjective experience of fatigue^44^. However, our study suggests that the PFC may be less vulnerable to the inflammatory insult from the tumor compared to the hypothalamus. This suggests that while the PFC is important for regulating fatigue-related behavior, the inflammatory pathways leading to CRF may initially activate other brain regions, like the hypothalamus, that are more vulnerable to peripheral inflammation. It remains to know if the relative resistance of PFC to inflammation from peripheral tumor is specific to the model or the tumor type. More studies should further clarify the brain-region and tumor type specificity.

By identifying the hypothalamus as a selectively affected site of tumor-induced neuroinflammation, our findings suggest a likely central node for further investigation into the CRF regulatory mechanism. Numerous studies have shown that the function of the HPA axis is associated with CRF^45^. The paraventricular nucleus of the hypothalamus serves as the central hub of the HPA axis and exerts its effects by producing CRH. Decreased CRH levels in chronic fatigue conditions such as CRF and chronic fatigue syndrome have been reported, suggesting that HPA axis hypofunction may contribute to fatigue symptoms through impaired stress adaptation, insufficient cortisol production, and sustained inflammation^46–48^. Conflicting findings have been noted in some models or conditions with acute stress^49^. These discrepancies may reflect differences in disease stage, fatigue subtype, or compensatory mechanisms within the HPA axis. In our study, we observed a hypofunctional HPA axis in the breast tumor-induced fatigue model, characterized by reduced CRH peptide expression in the hypothalamus and lower serum levels of CRH and cortisol. These results are consistent with previous reports implicating HPA axis dysregulation as a key neuroendocrine feature of CRF^50,51^. Interestingly, although CRH peptide levels were decreased, *CRH* gene expression levels in the hypothalamic tissue showed a significant increase in the Tumor group compared to the Con group, and this was normalized by PVSO treatment. This apparent discrepancy between mRNA and protein levels suggests a potential feedback regulatory mechanism at the transcriptional level in response to HPA axis deficiency. It also highlights the complexity of CRH regulation under tumor-related or inflammatory stress conditions. Such inconsistencies may arise from a range of post-transcriptional and post-translational regulatory processes, including mRNA modification, translation inhibition, or stress-induced protein degradation^52–54^. We plan to investigate these regulatory mechanisms in greater depth in future studies.

We here provided further evidence that the inflammation in the hypothalamus plausibly results in the reduction of CRH peptide levels, as activation of inflammation may inhibit the mRNA or protein synthesis via specific signaling^55^. The HPA axis operates through intricate feedback mechanisms and interacts closely with inflammatory pathways. In our study, in agreement with reduced CRH level in both the hypothalamus and serum, the serum cortisol level also decreased. Cortisol, a key anti-inflammatory hormone, plays a crucial role in regulating inflammation. Chronic cortisol deficiency can impair the function of the glucocorticoid receptor, thereby failing to regulate inflammatory pathways, i.e. activation of the NF-κB signaling cascade, leading to an enhanced release of pro-inflammatory cytokines^56,57^. We found the lack of change in ACTH levels, despite of the reduced levels for both CRH and cortisol. Similar findings have also been reported from other studies, likely due to chronic dysregulation of the HPA axis^58^. Nonetheless, our study suggests the immune response regulates the HPA axis via CRH neurons, and the precise mechanisms warrant further investigation.

Our study investigated the effects of peripheral inflammatory factors on the inflammation in the hypothalamus and HPA axis in the breast cancer, underlying the CRF. We showed that the improvement of CRF by PVSO may be attributed to the attenuation of the inflammation in the hypothalamus and thus restoration of the HPA axis. The mechanisms underlying tumor-induced hypothalamus inflammatory responses and their actions on the HPA axis for CRF require further investigation.

## Methods

### Animals

Male ICR or female BALB/c mice (Beijing Huafukang Biotechnology Co., Ltd.) at 4–5 weeks of age were housed at the Jinan University Laboratory Animal Center. All mice were placed under controlled standard barrier conditions (temperature 23 ± 2 °C, humidity 55 ± 5%, and 12-hour light-dark cycle at the Jinan University Animal Center) and had free access to food and water. All animal studies were conducted in accordance with the Jinan University Animal Experimentation Guidelines and were approved by the Jinan University Ethics Committee for the Care and Use of Animal Experiments (Approval Number: 20240411-01).

### Materials

The dietary supplement of *Ganoderma* spore oil (GSO) was produced by Guangzhou Hanfang Pharmaceutical Co., Ltd. (Guangzhou Hanfang), which was used as a positive control for relief of CRF^59^. Murine 4T1 mammary carcinoma cells were purchased from by Cell Bank, Chinese Academy of Sciences. LPS derived from Escherichia coli (O55:B5) was purchased in Merck SA (L2880, Darmstadt, Germany) and dissolved in saline for the construction of the fatigue model.

### Extraction of PVSO

The particles of *prunella vulgaris* seed powder were introduced into a kettle designed for supercritical CO_2_ extraction. A supercritical fluid consisting of CO_2_ with 0.3–3.5% ethyl acetate modifier was added to the kettle. The extraction process was carried out at a temperature of 28 ± 2 °C, a pressure of 38 ± 2 MPa, and a flow rate of 12–58 kg/h for a duration of 2 ± 0.5 h. The extraction procedure was conducted using a primary resolver at a pressure of 13 ± 1 MPa, and a secondary resolver at a pressure of 6 ± 1 MPa. A two-stage separation technique was employed, with the following process conditions: The first stage resolver had a pressure of 13 ± 1 MPa and a separation temperature of 36 ± 2 °C. The second stage resolver had a pressure of 6 ± 1 MPa and a separation temperature of 30 ± 1 °C. The Charcot seed was processed to obtain the oily extract, which was then subjected to separation and recycling of ethyl acetate to obtain crude Charcot seed oil. Finally, the crude oil was further processed to obtain the refined Charcot seed oil. PVSO contained a mass fraction of total unsaturated fatty acids exceeding 80%, a mass fraction of ω-3 unsaturated fatty acids exceeding 60% (with α-linolenic acid comprising over 50% of the total), a mass fraction of triterpenoids exceeding 5% (consisting of ursolic acid constituents), a negligible quantity of rosmarinic acid, and a diverse array of red fluorescence class point constituents.

### LPS-induced fatigue model and dosage-effect experiment

Based on the previous screening of higher doses of PVSO, we further conducted efficacy screening of PVSO at low and high doses of 0.13 g/kg (equivalent to 0.0208 g/kg in mice based on drug concentration). After a one-week acclimatization period, male ICR mice (6-8 weeks old) were randomly divided into five groups: Con group (Non-model Control), LPS group (LPS + saline oral administration), and three PVSO treatment groups with different doses (low-dose PVSO-L, medium-dose PVSO-M, and high-dose PVSO-H) in LPS treatment. PVSO-treated mice received PVSO via oral gavage at doses of low (0.0104 g/kg), medium (0.0208 g/kg), and high (0.0312 g/kg) doses, once daily for 7 days. Two days after PVSO treatment, mice received daily intraperitoneal injections of LPS (0.5 mg/kg, i.p) for five consecutive days and the Non-model Con group received injections of an equal volume of PBS.

Mouse body weight was monitored throughout the experiment. On day 7, the sucrose preference test (SPT) was initiated 0.5 h after oral administration. On day 8, the grip strength test (GST), OFT and TST were conducted sequentially. The intervals for all behavioral tests were at least for 2 h. On day 9, the RRT was performed. After the final behavioral testing, the mice were euthanized under deep anesthesia, and tissues were immediately harvested for subsequent histological, biochemical, and molecular biological analyses.

### Breast-tumor modeling experiment

The 4T1 cell cells were cultured in RPMI-1640 medium (Gibco, Shanghai, China) supplemented with 10% fetal bovine serum (Gibco, Shanghai, China), 100 U/ml penicillin, and 100 μg/ml streptomycin (Gibco, Shanghai, China). The culture was maintained at an incubation temperature of 37 °C and a humidity of 5%.

The female Balb/c mice were placed into four groups using random assignment, with 10 mice in each group. The groups were as follows: (1) control group (Con); (2) group with a tumor-bearing model by inoculation of 4T1 cells (Tumor); (3) Tumor with PVSO intervention group (PVSO); and (4) Tumor with GSO intervention (GSO). GSO is a commercial medicinal food, which has been shown to be effective to treat CRF, and thus used as a positive control. Mice in the Tumor, PVSO, and GSO groups were administered an injection of 100 μL of 4T1 cell suspension containing 1 × 10^5^ cells per mouse. Mice in the Con group were administered an injection of the same volume of PBS. The inoculation was administered subcutaneously on the right side of the body to prevent any disruption to future behavioral investigations following the inoculation. The day of 4T1 cell injection was considered as day 0. Starting from day 1, treatment with PVSO (0.0208 g/kg) and GSO (0.4 g/kg) was carried out while the mice in the Con and Tumor groups received equal volumes of solvent. Tumor size was monitored during this period using vernier calipers. The formula for tumor volume was as follows: V = (A × B^2^)/2 mm^3^, and the tumor volume was calculated by measuring the maximum diameter (A) and vertical diameter (B) of the tumor. Twenty-four hours after the last gavage, mice were subjected to behavioral tests and then euthanized. A schematic of the experimental design is shown in Fig. 2A.

### Grip strength test

Ahead to the actual trial, a preliminary training session was conducted to acquaint the mice with the grip test. During the formal experiment, the mice were held by their tails and allowed to naturally grip the force meter. Their tails were gently pulled apart in parallel until the maximum pulling force was reached, at which point their claws were released. The readings on the grasping force meter were recorded and multiple measurements were taken to ensure the accuracy and credibility of the results.

### Rotarod test

The mice were placed on a stationary rod and given 5–10 min to get used to their surroundings. Then, the speed of the rod was gradually increased to a level that the mice could handle, and they were trained for another 5–10 min. Multiple trials were conducted. During the formal experiment, the mice were positioned on a rotating rod, and the rotational velocity of the rod was systematically raised during the testing phase. Data, such as the time at which the mice fell or lost their equilibrium within the 5–10 min interval, were meticulously documented. Several tests were conducted.

### Tail suspension test

The tails of the mice were secured using adhesive tape, positioned 1 cm from the tip of the tail. The mice were then suspended from the rod of the box, with their heads kept approximately 50 cm from the horizontal. An opaque partition was used to separate the heads for videotaping. Each mouse was observed for a total of 6 min, during which the cumulative time of immobility for the last 4 min was recorded. The total duration of immobility for each mouse was measured in seconds.

### Open field test

The mice were confined within a flat, enclosed area. The mine field reaction area was comprised of four reaction boxes, each measuring 50 cm × 50 cm × 40 cm. These containers had black-painted interiors and were equipped with cameras on top. The cameras automatically monitored and recorded the mice’s movements. The reaction box was divided into a central and peripheral area. Prior to the start of the experiment, the mine field box was wiped clean with alcohol, the mice were placed in the central zone, each mouse was placed in the same direction, and after placing as, the experimenter left the test area and began to record the amount of time that the mice stayed in the central zone and the cumulative distance traveled during the 5-minute period.

### Sucrose preference test

The mice had a training period when they were exposed to a 2% sucrose solution for 24 h before the SPT experiment. This was done by placing two bottles holding the solution in their cage. Subsequently, the contents of one of the bottles were substituted with water, while the placements of the two bottles were interchanged for a duration of 24 h. Over the past 24 h, the mice underwent a 12-hour period of water and food deprivation. Following this, two bottles were provided: one containing water and the other with a 2% sucrose solution. The mice were allowed unrestricted access to both bottles. Fluid consumption was measured throughout a 6-hour timeframe. Sucrose preference was determined by calculating the ratio of sucrose solution consumption to the total consumption of both sucrose solution and water. The criterion for lack of pleasure in the experimental group was defined as a significant decrease in the sucrose preference ratio compared to the control group in the preference test^60,61^.

### Euthanasia and sample collection

After behavioral testing, mice that had been fasted for 12 h were given an intraperitoneal injection of sodium pentobarbital (50 mg/kg bw), and the absence of reflexes (e.g., stirrup reflex and corneal reflex) confirmed that the mice were unconscious. After deep anesthesia, blood samples were taken from the retro-orbital plexus of the mice, centrifuged at 3000 × g for 10 min, and the serum was extracted and stored at −80 °C. The mice were then subjected to a cervical dislocation. All operations were performed in strict accordance with ethical guidelines and approved by the Institutional Committee for the Care and Use of Animals. Brain tissues were subsequently dissected, and hippocampus, prefrontal cortex and hypothalamus tissues were removed for preservation. All serum samples and tissue samples were stored in a refrigerator at −80 °C.

### Hematoxylin and eosin (H&E) staining

After behavioral testing, mice were deprived of food for 12 h and anesthetized by intraperitoneal injection of pentobarbital sodium at a dose of 50 mg/kg, then tumors were stripped from the mice and size and weight were recorded. Tumors were dehydrated in sucrose solution in a gradient, followed by addition of embedding agent for embedding, and frozen at −80 °C. The frozen sections were taken out from the −20 °C refrigerator and returned to room temperature, fixed with tissue fixative for 15 min, and then rinsed with running water. Sections were put into the HD constant dye pretreatment solution for 1 min, hematoxylin staining solution for 3–5 min, differentiation solution for differentiation, and return to blue solution for return to blue, and rinsed with running water after each step. Then the sections were dehydrated in 95% alcohol for 1 min and stained in eosin staining solution for 15 s. Finally, the sections were dehydrated and sealed.

### Immunofluorescent staining experiment

Frozen section is baked in oven at 37 °C for 10–20 min, and moisture is controlled. Fixed in fixed solution for 30 min, washed in PBS (PH7.4) on decolorizing shaking table for 3 times, 5 min each time. After repair, cool naturally. The slide was placed in PBS (PH7.4) and washed by shaking on the decolorizing shaker for 3 times, 5 min each time. After the section is slightly dried, draw a circle around the tissue with a tissue pen, add BSA, and seal it for 30 min. Drop the prepared primary antibody and place the slices horizontally in a wet box at 4 °C for overnight incubation. The primary antibodies as following were used: Anti-KI67 (GB111499, 1:500 dilution) antibody was purchased from Servicebio (Wuhan, China). Then add the corresponding secondary antibody and incubate at room temperature for 50 min away from light. After the above two steps, the slides were washed in PBS by shaking on a decolorizing shaker three times for 5 min each time. Add DAPI dye solution (G1012, Servicebio, Wuhan, China) and incubate at room temperature away from light for 10 min. Add quenching agent B solution for 5 min and rinse with water for 10 min. Finally, seal the slide with an anti-fluorescence quencher.

### Western blotting

Western blotting was performed to detect the protein level in hypothalamic tissues. Hypothalamic tissue samples were added to lysis buffer and centrifuged at 15,000 × g at 4 °C. The protein supernatant was then extracted, and the protein concentration was measured by the BCA method. Subsequently, gel preparation, loading, electrophoresis, and membrane transfer were performed; 5% skim milk powder and bovine serum protein were enclosed for 2 h, and the primary antibody was incubated at 4 °C overnight. Then, the membranes were washed twice with TBST for 5 min each time, and the secondary antibody (K1223, APExBIO, Houston, USA) was incubated for 2 h at 4 °C. The primary antibodies were applied as following: anti-NF-κB (#10745-1-AP, 1:3000 dilution), anti-CRH (#10944-1-AP, 1:3000 dilution) and Anti-β-actin (#66009-1-Ig, 1:10000 dilution) antibodies was obtained from Proteintech (Wuhan, China). Anti-phospho-NF-κB antibody (#3039, 1:3000 dilution) were purchased from Cell Signaling Technology (Boston, MA, USA). Finally, the membranes were incubated in ECL luminescent solution (G2020, Servicebio, Wuhan, China) for 3–5 min, subjected to darkroom exposure, and the bands were scanned, grayscale values were calculated, and analyzed and data.

### qRT-PCR

The RNA from hypothalamic tissue was converted into complementary DNA (cDNA) using the HiScript III RT SuperMix for qPCR (+gDNA Wiper) kit. The ChamQ Universal SYBR qPCR premix kit was then used to conduct quantitative reverse transcription polymerase chain reaction (qRT-PCR), with GAPDH serving as the reference gene. PCR reaction system volume is 20 μL. The PCR reaction system (20 μL) comprised of 10 μL of 2 × ChamQ Universal SYBR qPCR premix, 0.4 μL of forward primer (10 μM), 0.4 μL of reverse primer (10 μM), 0.5 μL of template DNA, and 8.7 μL of ddH2O. The PCR reaction was conducted at a temperature of 95 °C and 1.5 °F using a ChamQ Universal SYBR qPCR premix kit (20 μL). The Thermo Fisher USA product should be pre-denatured at a temperature of 95 °C for a duration of 30 s. This should be followed by 40 cycles of 95 °C for 10 s and 60 °C for 30 s. The lysis curve collecting process involved heating at 95 °C for 15 s, followed by cooling at 60 °C for 1 min, and then heating again at 95 °C for 15 s. The mean cycling threshold (Ct) was acquired for data analysis. The expression was determined using the 2^-ΔΔCt^ technique.

### Serum TNF-α and IL-1β, CRH, adrenocorticotropic hormone (ACTH) and cortisol levels

Serum was collected by centrifugation at 12,000 g for 10 min, and the serum levels of TNF-α, IL-1β, CRH, ACTH and cortisol were measured in each group of mice according to the instructions of the ELISA kit (Shanghai Jianglai Biotechnology Co., Ltd).

### Statistical analysis

SPSS 22 (SPSS, Inc.) and Graphpad Prism 8.0 software were used for statistical analysis. Statistical analysis was conducted only for studies in which each group had a sample size (n) of at least 5. One-way ANOVA was performed, followed by Bonferroni’s post hoc test for multiple comparisons if the F-test indicated significance (*P* < 0.05) and assumptions of normality and homogeneity of variance were met. Two-way repeated-measures ANOVA with post hoc comparison were used for all time-dependent experiments. A *P*-value of less than 0.05 was considered statistically significant for all analyses.

## Supplementary information

Supplementary data

## Data Availability

No datasets were generated or analysed during the current study.
