# Supplementary material for: Prunella vulgaris seed oil alleviates cancer related fatigue through hypothalamic inflammation and CRH regulation
Source: NPJ Sci Food. 2025 Sep 24;9:194. doi: 10.1038/s41538-025-00541-5 (PMC12460840; doi:10.1038/s41538-025-00541-5)
Supplement: Supplementary file 1 — Supplementary data [file 41538_2025_541_MOESM1_ESM.doc]

**Supplemental figures**

**
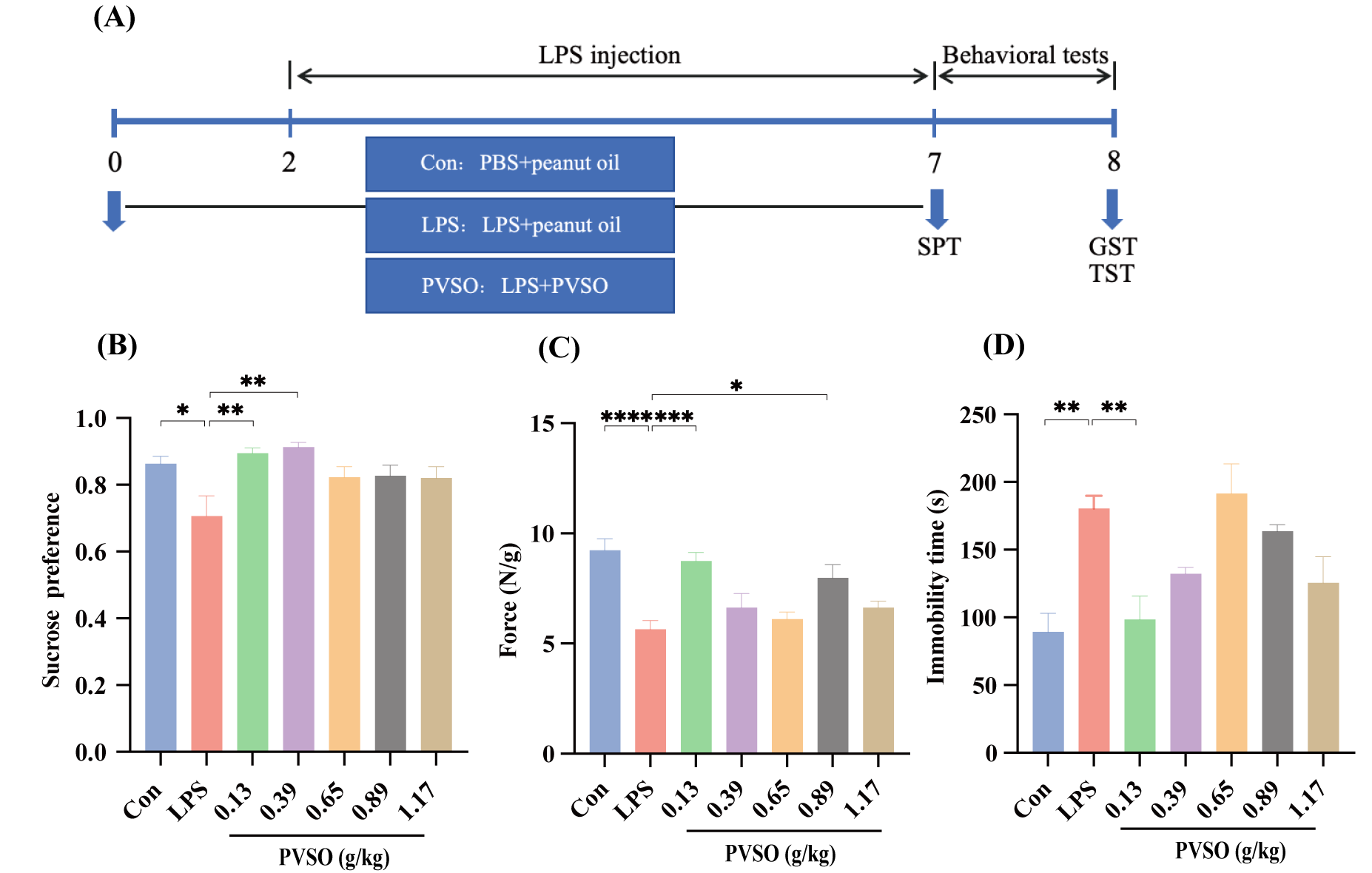
**

**Figure S1.** Dose-pharmacodynamic relationship of PVSO. (A) Experimental timeline: Mice were administered PVSO (0.13, 0.39, 0.65, 0.89, or 1.17 g/kg, dissolved in peanut oil) or vehicle (peanut oil) for 8 consecutive days. On day 3, LPS (0.5 mg/kg, i.p.) or PBS was injected to induce fatigue-like phenotypes. Behavioral tests were conducted on days 7 and 8. (B) Sucrose preference index of Sucrose preference test (SPT). (C) Grip strength magnitude in the grip strength test (GST). (D) The immobility time of the tail suspension test (TST). Data are expressed as mean ± SEM. **p* < 0.05, ***p* < 0.01 by One-way ANOVA (n = 8 per group).

**
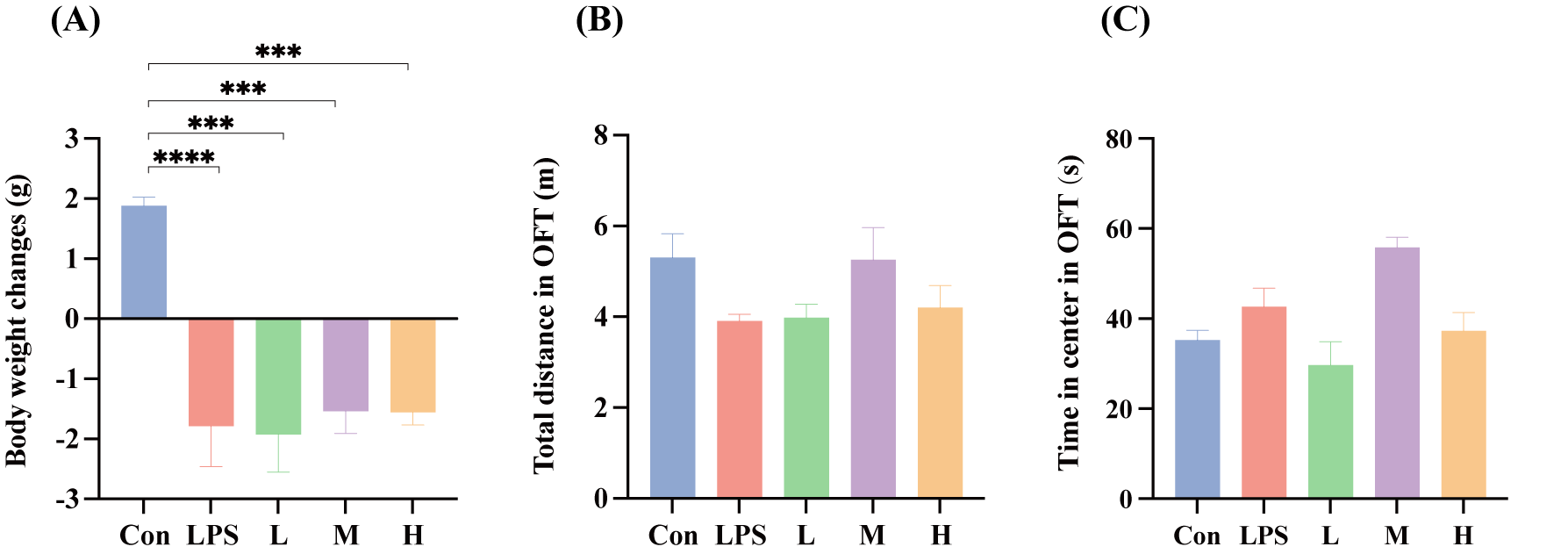
**

**Figure S2.** The effects of PVSO on body weight and open field behavior in LPS-induced fatigue model mice. (A) Changes in body weight of mice in each group one week after LPS or PBS injection. (B) Total distance traveled in the open field test (OFT) and (C) time spent in the central region of the OFT. Data are presented as mean ± SEM. **p* < 0.05, ***p* < 0.01 compared by One-way ANOVA(n = 6 per group).


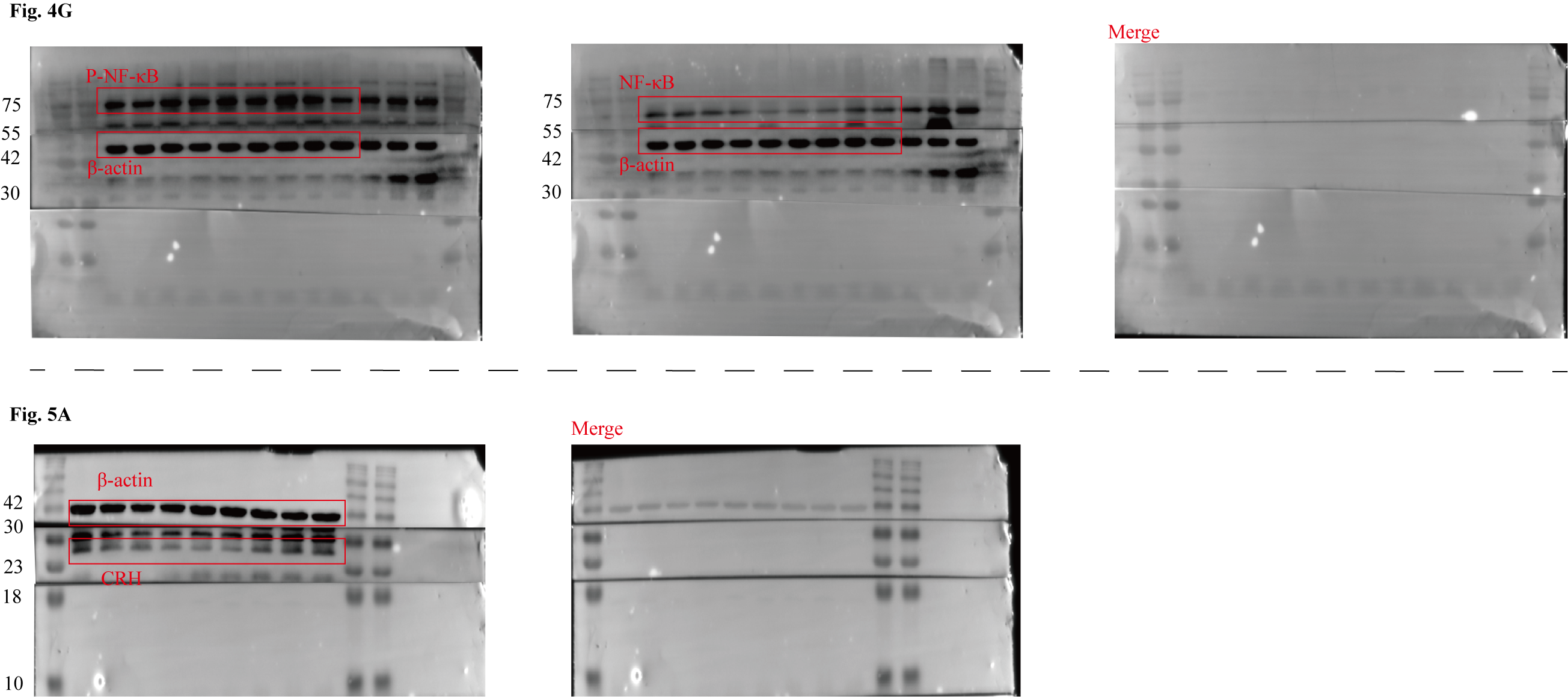


**Figure S3.** Original blots for Fig. 4G and 5A.

**Supplemental table**

**Table S1 Sequences of the PCR primers**

| Gene | F/P | Sequence |
| --- | --- | --- |
| *GAPDH* | Forward Primer | ACTCCACTCACGGCAAATTCAAC |
|  | Reverse Primer | ACACCAGTAGACTCCACGACATAC |
| *IL-1β* | Forward Primer | TCGCAGCAGCACATCAACAAG |
|  | Reverse Primer | CACCAGCAGGTTATCATCATCATCC |
| *TNF-a* | Forward Primer | CACGCTCTTCTGTCTACTGAACTTC |
|  | Reverse Primer | CTTGGTGGTTTGTGAGTGTGAGG |
| *NF-κB* | Forward Primer | ATCATCCACCTCCACGCTCAG |
|  | Reverse Primer | TCCTCTACTACATCTTCCTGCTTGG |
| *IL-6* | Forward Primer | GAGAGGAGACTTCACAGAGGATACC |
|  | Reverse Primer | TCATTTCCACGATTTCCCAGAGAAC |
| *CRH* | Forward Primer | TCTGTCGTCCTGCCTGCCTTG |
|  | Reverse Primer | CTTCACCCATGCGGATCAGAACC |
